# Exploring the link: Systemic immune-inflammation index as a marker in endometriosis—Insights from the NHANES 2001–2006 cross-sectional study

**DOI:** 10.1371/journal.pone.0304591

**Published:** 2024-06-06

**Authors:** Liang Peng, Xiaohan Luo, Baodi Cao, Xiaohui Wang

**Affiliations:** The Second People’s Hospital of Jingdezhen, Jingdezhen, Jiangxi, China; Dipartimento di Scienze Mediche e Chirugiche (DIMEC), Orsola Hospital, ITALY

## Abstract

**Objective:**

The systemic immuno-inflammatory index (SII), a novel immune marker of inflammation, has not been previously associated with endometriosis. The objective of this research is to explore the link between SII and the occurrence of endometriosis.

**Methods:**

Utilizing data from the National Health and Nutrition Examination Survey (NHANES) spanning 2001 to 2006, we screened and extracted relevant information from the population. Participants missing data on either SII or endometriosis were excluded. We divided the remaining cohort into quartiles based on SII levels: Q1 (SII < 249, n = 848), Q2 (249 ≤ SII < 604.55, n = 847), Q3 (604.55 ≤ SII < 825.35, n = 847), and Q4 (SII ≥ 852.35, n = 848). Multiple linear regression and smooth curve fitting techniques, were to evaluate the non-linear association between SII and endometriosis.

**Results:**

The study included 3,390 adults aged 20 to 55. Multiple linear regression analysis revealed a significant positive correlation between SII and endometriosis [3.14, 95% CI (2.22, 4.45), *P* < 0.01]. This correlation was consistent across subgroups defined by marital status, poverty income ratio, BMI, alcohol consumption, and age at first menstrual period. However, the relationship between SII and endometriosis was significantly modified by age, education, and history of pregnancy in the stratified analyses. The curve fitting indicated an S-shaped curve, with an inflection point at SII = 1105.76.

**Conclusion:**

The SII may serve as a predictive marker for endometriosis risk among women in the United States, offering a potentially simple and cost-effective approach. However, given the cross-sectional design of this investigation, further validation in prospective studies is necessary.

## Introduction

Endometriosis, a chronic ailment affecting women of reproductive age, is characterized by ectopic endometrial tissue growth in non-uterine sites, such as ovaries, the pelvic cavity, and the peritoneum [1]. This disorder not only triggers severe pain and irregular menstruation but can also lead to infertility [2]. Recent studies have posited a potential link between endometriosis and several factors, including hormonal imbalances, immune system dysfunctions, and genetic predispositions [3]. Raimondo D et al. [4] highlight central sensitization as a key factor, noting its significant prevalence among women with endometriosis. Treatment options typically encompass medications, hormone therapy, and surgical interventions. Given the disease’s recurrent and chronic nature, management often demands a tailored, long-term strategy [5]. Additionally, research has illuminated a notable association between endometriosis and mental health complications, thereby accentuating the necessity for a comprehensive treatment paradigm [6, 7]. Enhanced understanding and effective management of endometriosis are crucial in advancing women’s health outcomes.

Endometriosis, characterized as both an inflammatory and immune-related disease, presents a pro-inflammatory state within the endometrial immune environment, potentially impacting embryo implantation and disease progression [8]. The role of immune cells in the development of endometriosis, especially the influence of endocrine-immunological interactions on its progression, has become a focal point of research [9]. Recent studies emphasize the link between systemic immune-inflammatory states and endometriosis, noting a higher incidence in young women with autoimmune or inflammatory diseases [10], and an elevated allergy prevalence among endometriosis patients [11]. Immune cell counts, reflecting systemic immune and inflammatory status, appear to correlate with endometriosis risk. For example, endometriosis patients exhibit a reduction in the levels of classical and intermediate monocytes, while the levels of plasmacytoid dendritic cells and non-classical monocyte increase in the blood [10]. Additionally, lower iTreg and Treg/Th17 ratios and higher Th17 (inflammatory) levels have been observed in patients not receiving hormone therapy compared to those who are [12]. These findings underscore the importance of exploring new inflammation-based or immune cell count-related markers for endometriosis risk assessment, crucial for prevention strategies.

Introduced by Hu et al. in 2014 [13], the systemic immune-inflammation index (SII) consolidates counts of neutrophils, lymphocytes, and platelets into a formula for assessing systemic immune-inflammatory status. SII has emerged as a simple, cost-effective, and reliable clinical index, useful in diagnosing and managing various diseases. Notably, higher SII values in hepatocellular carcinoma patients have been linked to poorer overall and disease-free survival [14], with similar patterns observed in other solid tumors like esophageal, ovarian, and pancreatic cancers [15–17]. Beyond its applications in oncology, the SII has also been validated for cardiovascular disease risk assessment [18] and in the context of autoimmune diseases and chronic inflammatory such as chronic obstructive pulmonary disease (COPD) [19, 20]. Despite its widespread clinical utility, the relationship between SII and endometriosis remains relatively unexplored. This study aims to elucidate the correlation between SII levels and endometriosis among participants from the National Health and Nutrition Examination Survey (NHANES) in the United States, with the objective of offering valuable insights for endometriosis prevention.

## Methods

### Collection of research data

**Fig 1:**
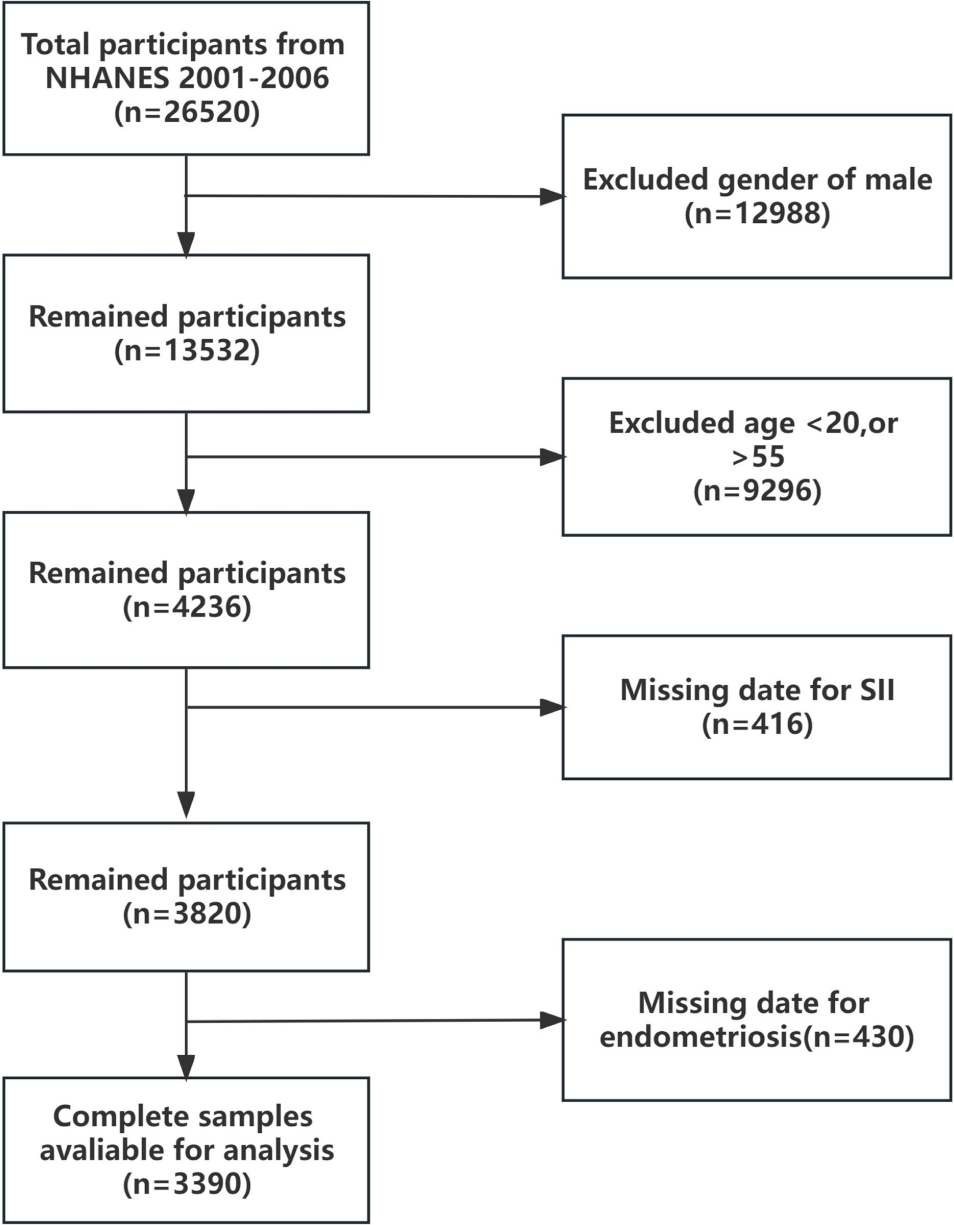
Flowchart of the participant selection process.

The data utilized in this study were sourced from the NHANES database, an extensive and nationally representative survey initiative launched by the National Center for Health Statistics (NCHS) in 1999. NHANES gathers a wide array of health-related information using methods including questionnaire-based interviews, physical examinations, and laboratory tests. It employs a sophisticated multistage probability sampling design to amass diverse health-related data. Participants are interviewed at their homes and subsequently undergo physical examinations and laboratory evaluations at a Mobile Examination Center (MEC), making NHANES a crucial resource for assessing the health and nutritional status of the U.S. population and supporting epidemiological and health science research. Ethical approval for NHANES research protocols was granted by the Research Ethics Review Board of the National Center for Health Statistics (NCHS), under Protocol #2005–06, securing written informed consent from every participant. For this study, all analyses were meticulously conducted in accordance with NHANES guidelines and regulations. Through an exhaustive search and screening of the NHANES database from 2001 to 2006, we selected female participants aged 20–55 years, excluding males and individuals with incomplete information on ’self-reported endometriosis’, ’SII’, and ’covariates’, resulting in a final cohort of 3,390 participants. Fig 1 illustrates the screening process flowchart.

### Dependent and independent variables

In this study, data on endometriosis were obtained from the Reproductive Health Questionnaire (RHQ360) and the Reproductive Health Diagnosis (RHD361) self-report sections of the NHANES, where participants were queried on whether they had been diagnosed with endometriosis by a healthcare professional. Individuals confirming such a diagnosis were classified into a group representing self-identified cases of endometriosis, whereas those who negated the diagnosis formed the control group, indicating an absence of self-reported endometriosis.

The primary independent variable, the SII, is a novel inflammation marker calculated by multiplying platelet and neutrophil counts and then dividing by the lymphocyte count. To derive the SII, data from NHANES’s "L25" and "Complete Blood Count (CBC)" segments were utilized, specifically employing lymphocyte (LBDLYMNO), neutrophil (LBDNENO), and platelet (LBXPLTSI) counts. These values were then used to calculate the SII for each participant, which was further categorized into four quartiles (Q1 to Q4) for analysis.

### Covariate

Variables including age, ethnic background, marital status, educational attainment, and economic status, determined via interviews in households, were incorporated into the study’s analysis. Age was stratified into three groups: 20 to 29 years (Group 1), 30 to 39 years (Group 2), and 40 to 55 years (Group 3). Marital status categories were married, divorced, widowed, separated, never married, or living with a partner. Educational attainment was classified as either below high school or high school and above. Economic status were gauged using the poverty income ratio (PIR), with classifications being low (PIR < 1.4), middle (1.4 ≤ PIR ≤ 3.0), and high (PIR > 3.0). Alcohol consumption was classified based on annual drink count: non-drinker (0 drinks), light drinker (1–30 drinks), or heavy drinker (>30 drinks). Disease-related covariates included age of menarche and pregnancy history, sourced from RHQ010 and RHQ031. Body mass index (BMI) classifications were established as underweight (BMI < 25kg/m^2^), normal weight (25 kg/m^2^ ≤ BMI ≤ 30 kg/m^2^), and overweight (BMI > 30 kg/m^2^).

### Statistical analysis

The baseline characteristics of participants were detailed utilizing descriptive statistical methods: mean values and standard deviations were used for continuous variables, and categorical variables were described using percentages.

The relationship between SII and endometriosis was analyzed using multifactorial logistic regression. Three models were employed in the analysis: Model 1, which was unadjusted, Model 2, which took into account adjustments for age, ethnicity, educational background, and poverty-income ratios, and Model 3, which included comprehensive adjustments for all covariates. Weighted one-way logistic regression assessed the impact of covariates on the SII-endometriosis association. Statistical analyses were performed with R software, version 4.3.2 (accessible at http://www.R-project.org). In these analyses, a two-tailed *P*-value below 0.05 was considered statistically significant.

## Results

### Baseline characteristics

Table 1 presents an overview of the initial characteristics of the 3,390 individuals participating in the study. Among these, 3,063 were not diagnosed with endometriosis, whereas 327 were. On average, participants were 35.72 years old. The average age among those diagnosed with endometriosis was 38.60 years, in contrast to 35.41 years in those without, suggesting an increased incidence in older individuals. Moreover, endometriosis prevalence was higher among married, widowed, divorced, or separated individuals. In the group with endometriosis, the BMI averaged 28.61 kg/m^2^, while it was slightly higher at 29.89 kg/m^2^ in the group without endometriosis. However, with a *P*-value above 0.05, there was no discernible significant link between BMI and the occurrence of endometriosis. Similar trends were noted in alcohol consumption and pregnancy history. Significant associations with endometriosis were found for race, education, household income poverty ratio, and age of menarche. More specifically, non-Hispanic whites, individuals with at least a high school education, those with higher household income poverty ratios, and those with a lower mean age of menarche, were more prevalent among endometriosis cases. Blood analysis showed higher mean neutrophil and platelet counts, but lower lymphocyte counts in endometriosis patients, with a statistically significant *P*-value of less than 0.05.

**Table 1:** Baseline characteristics of participants (N = 3390).

	Total (N = 3390)	Non-endometriosis (N = 3063)	Endometriosis (N = 327)	*P*-value
**Age(year)**	35.72 ± 10.11	35.41 ± 10.12	38.60 ± 9.59	<0.001
**Race**				<0.001
Mexican	747(22.04%)	709 (23.15%)	38 (11.62%)	
Other Hispanic	134(3.95%)	125 (4.08%)	9 (2.75%)	
White	1627(47.99%)	1421 (46.39%)	206 (63.00%)	
Black	724(21.36%)	662 (21.61%)	62 (18.96%)	
Other Race	158(4.66%)	146 (4.77%)	12 (3.67%)	
**Ratio of family income**	2.66 ± 1.63	2.63 ± 1.62	2.90 ± 1.68	0.010
**Education**				0.002
Below high school	748(22.06%)	698 (22.79%)	50 (15.29%)	
High school and above	2642(77.94%)	2365 (77.21%)	277 (84.71%)	
**Marital Status**				0.009
Married	1877(55.37%)	1690 (55.17%)	187 (57.19%)	
Widowed	49(1.45%)	42 (1.37%)	7 (2.14%)	
Divorced	300(8.85%)	257 (8.39%)	43 (13.15%)	
Separated	134(3.95%)	120 (3.92%)	14 (4.28%)	
Never married	691(20.38%)	642 (20.96%)	49 (14.98%)	
Living with partner	339(10.00%)	312 (10.19%)	27 (8.26%)	
**BMI(**kg/m^2^)	28.86 ± 7.38	28.89 ± 7.44	28.61 ± 6.90	0.732
**Age of menarche**	12.58 ± 1.73	12.60 ± 1.72	12.35 ± 1.81	0.015
**Pregnancy history**				0.126
Yes	2844(83.89%)	2560 (83.58%)	284 (86.85%)	
No	546(16.11%)	503 (16.42%)	43 (13.15%)	
**Alcohol(number of cups)**	2.90 ± 12.82	2.81 ± 11.62	3.80 ± 20.95	0.099
**LYMP(1000/mL)**	2.18 ± 0.68	2.20 ± 0.68	2.04 ± 0.70	<0.001
**NEUT(1000/mL)**	4.84 ± 2.06	4.75 ± 1.94	5.71 ± 2.80	<0.001
**PLT(1000/mL)**	290.53 ± 72.11	289.66 ± 72.13	298.70 ± 71.64	0.019
**SII**	687.38 ± 378.43	659.85 ± 327.39	945.28± 639.27	<0.001

### Multiple regression model results

**Fig 2:**
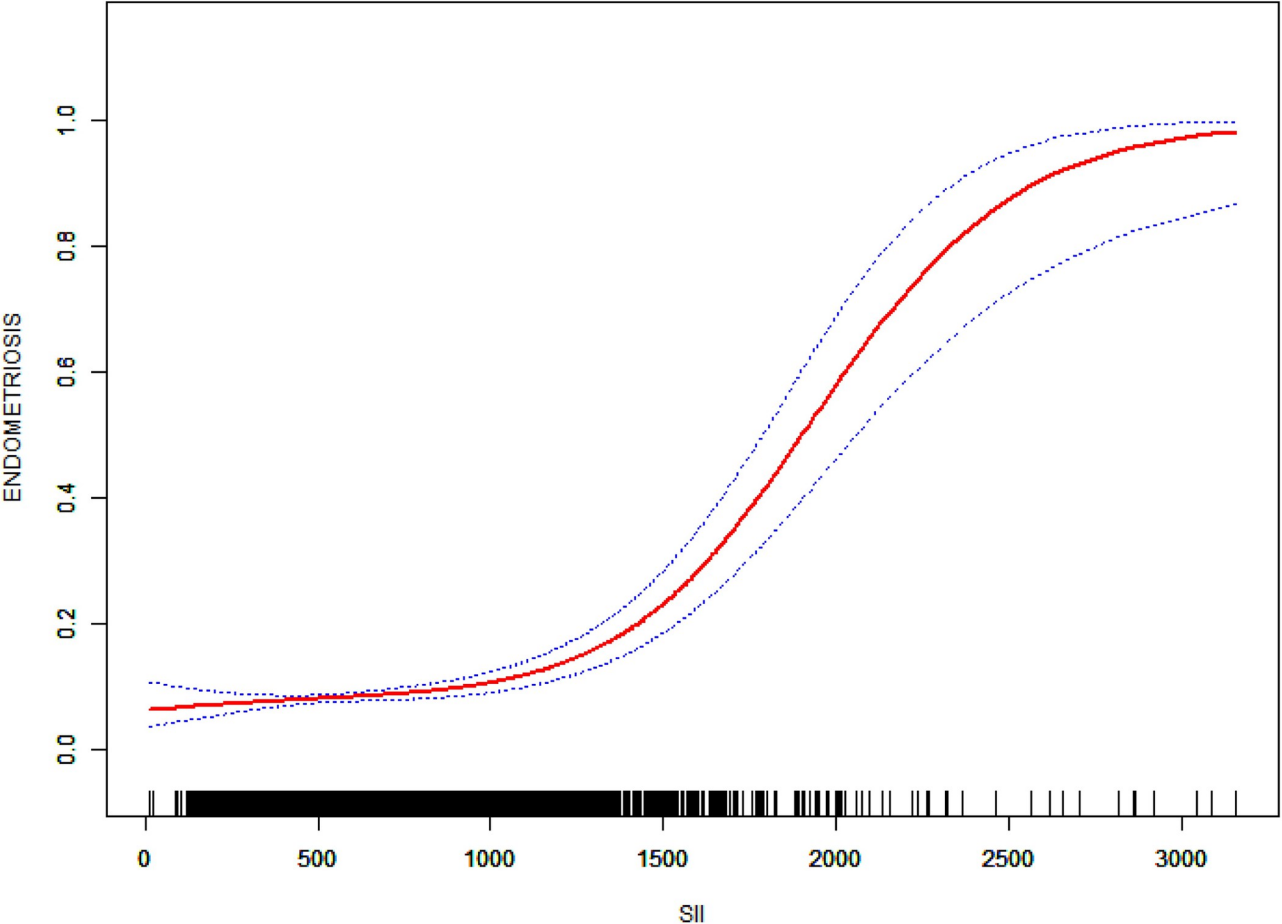
The correlation between SII and endometriosis rate.

A multifactorial logistic regression approach was employed to investigate the association between SII and the incidence of endometriosis. As presented in Table 2, the stepwise construction of three models established a statistically significant positive correlation between SII levels and endometriosis prevalence in Model 3. Specifically, for each unit increase in SII within the uppermost quartile (Q4), there was a 2.14% rise in the probability of having endometriosis relative to the bottom quartile (Q1). The regression curve in Fig 2, demonstrating an "S" shape, revealed a positive correlation between increased SII levels and endometriosis risk. Through threshold effect analysis, a critical point an SII value of 1105.76 was identified; beneath this benchmark (*K* < 1105.76), the correlation was statistically non-significant (*P* = 0.2205), whereas beyond it (*K* > 1105.76), a significant positive correlation was noted.

**Table 2:** Multivariate logistics regression model of SII infection and endometriosis.

	Model 1	Model 2	Model 3
**SII**			
Q1	1.0	1.0	1.0
Q2	1.23 (0.85, 1.76)	1.24 (0.86, 1.80)	1.26 (0.87, 1.83)
Q3	1.11 (0.77, 1.61)	1.20 (0.82, 1.75)	1.23 (0.84, 1.80)
Q4	2.58 (1.87, 3.57)	3.01 (2.14, 4.23)	3.14 (2.22, 4.45)
***P* for trend**	<0.001	<0.001	<0.001

Note: In this study, endometriosis was examined as the dependent outcome, with the Systemic Immune-Inflammation Index (SII) concentration being analyzed as an independent factor. To investigate the relationship between SII and endometriosis, multivariate logistic regression analyses were conducted, considering a range of potential confounding factors. Model 1, the initial model, did not include adjustments for any potential confounders. Model 2 adjusted for variables such as participant age, marital status, poverty-to-income ratio, and race. Model 3 further refined these adjustments to include all covariates, offering a comprehensive analysis.

### Subgroup analysis

**Fig 3:**
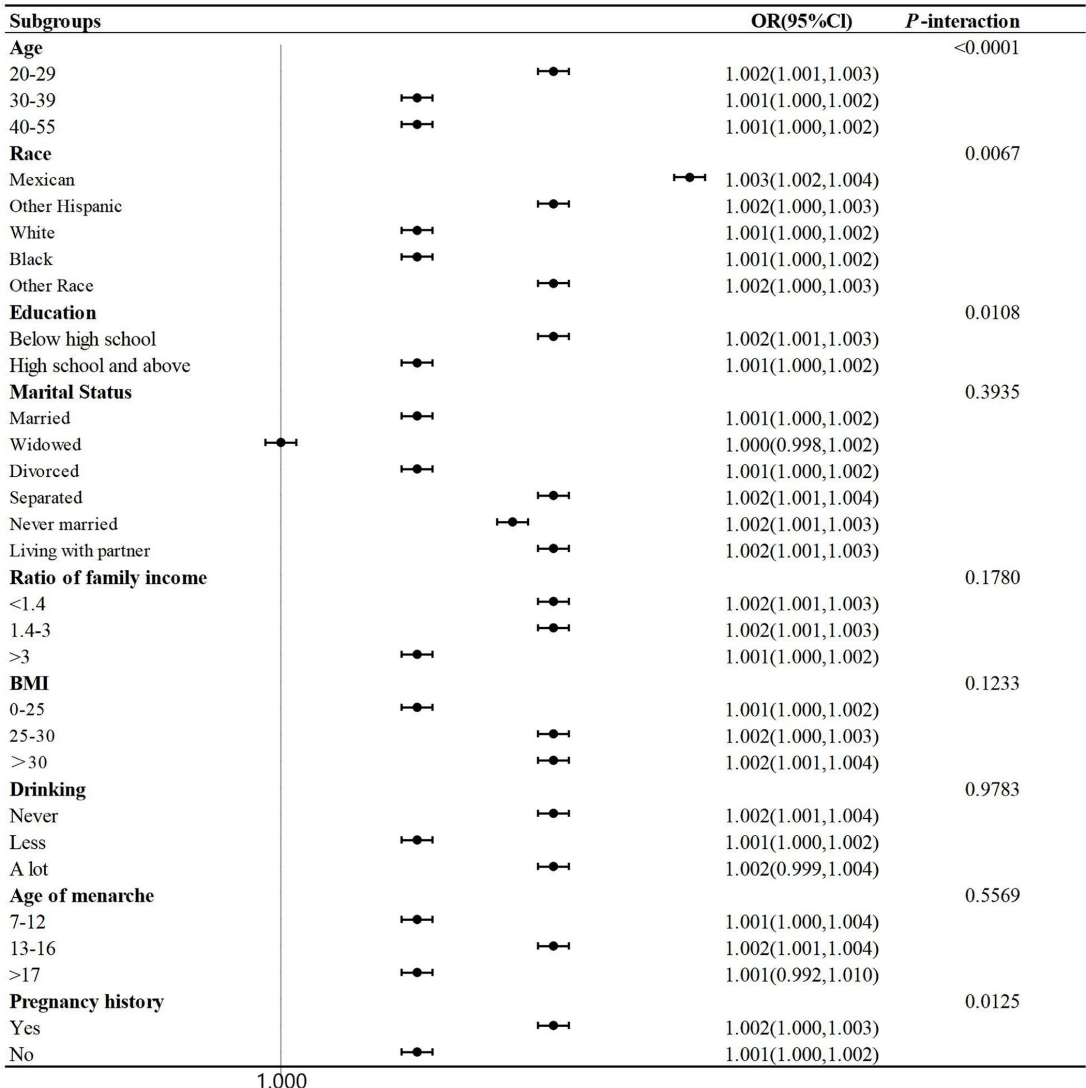
Subgroups analyses of the effect of SII on endometriosis.

Subgroup analyses and interaction tests were conducted to assess the consistency of the association between SII and endometriosis across various demographic characteristics. Stratification was based on age, race, education, marital status, poverty income ratio, alcohol consumption, BMI, age of menarche, and pregnancy history. As shown in Fig 3, there were no significant interactions between SII and marital status (*P* = 0.3935), poverty income ratio (*P* = 0.1780), BMI (*P* = 0.1233), alcohol consumption (*P* = 0.9783), and age of menarche (*P* = 0.5569). However, age (*P* < 0.001), race (*P* = 0.0067), educational level (*P* = 0.0108), and pregnancy history (*P* = 0.0125) significantly influenced the relationship between SII and endometriosis.

## Discussion

This study conducted a thorough data analysis of 3,390 participants from the NHANES database, uncovering a positive correlation between endometriosis risk in women and SII levels. Subsequent analysis revealed an S-shaped curve relationship between SII levels and endometriosis incidence, with a critical point at SII = 1105.76. For *K*<1105.76, the *P* value denoting the association between SII and endometriosis was 0.2205, signifying no statistical significance; conversely, for *K*>1105.76, a statistically significant and positive correlation emerged.

To our knowledge, this study is the first cross-sectional analysis to assess the link between SII and endometriosis. It demonstrated that, compared to SII levels in the first quartile (Q1), each unit increase in SII at the fourth quartile (Q4) heightened the probability of endometriosis by 2.14%. The study by M M Li et al. [21], which contrasted other benign ovarian cysts (n = 150) with ovarian endometriomas (a form of endometriosis, n = 350), observed a higher median SII in the latter group (median: 488.70), though without notable statistical difference. It is worth mentioning that M M Li et al.’s research also reported the predictive value of SII for postoperative recurrence of ovarian endometriomas (AUC = 0.803). Unlike M M Li et al.’s retrospective analysis, our large-scale, population-based cross-sectional study established a positive correlation between SII levels and endometriosis occurrence. Furthermore, subgroup analyses indicated that marital status, economic status, BMI, alcohol consumption, and age of menarche did not markedly affect the SII-endometriosis relationship. However, age, race, education level, and pregnancy history were found to significantly influenced the relationship between SII and endometriosis. The study by Feng J et al. suggests that in China, endometriosis prevalence increases before the age of 24 and decreases with age thereafter [22]. Shafrir AL et al. reports that a higher number of births is associated with a lower risk of endometriosis [23]. Compared to White women, Black women have a lower likelihood of being diagnosed with endometriosis [24]. Although previous research has not shown a significant relationship between education level and endometriosis [25], our study reveals that education level significantly influences the association between SII and endometriosis. This discovery necessitates additional investigation.

The exact mechanisms linking inflammation, immunity, and endometriosis remain unclear. Endometriosis is associated with an inflammatory response that can cause endothelial dysfunction and might even lead to carcinogenesis [26]. The SII’s association with endometriosis likely stems from its reflection of the body’s inflammatory-immune state, with high levels indicating increased inflammation and a weakened immune response. Ottolina J et al. [27] found higher neutrophil counts and lower lymphocyte counts in endometriosis patients, aligning with studies on neutrophils’ role in early endometriosis formation [28]. Recent research has linked neutrophil infiltration in ovarian-type endometriosis lesions with disease severity and has found that these lesions induce neutrophils to express PD-L1, thereby inhibiting CD8+ T cell activity [29]. Similarly, platelets, akin to neutrophils, appear to suppress immunity within endometriotic foci. Elevated platelet aggregation and activation were observed in endometriosis patients, with platelet-derived TGF-β1 reducing NK cell cytotoxicity [30]. The roles of various lymphocyte subsets, including B cells, T cells, and NK cells, introduce complexity, as their precise roles in endometriosis remain under investigation. A notable reduction in the subsets of lymphocytes characterized by CD25 and CD3 markers in the peritoneal fluid of patients with endometriosis [31]. A meta-study reported no significant difference in peripheral blood CD4+ T cell levels between mild and severe endometriosis cases, though there was a noted decrease in CD4+ T cell levels within the peritoneal fluid of patients [32]. Additionally, elevated peripheral blood NK cell percentages were noted in advanced endometriosis cases, specifically affecting the rectosigmoid colon [33]. Thus, the increased risk of endometriosis associated with higher SII may be linked to the activities of neutrophils, lymphocytes, and platelets.

Our findings propose SII as a direct, non-invasive marker for predicting endometriosis risk. However, well-established inflammatory markers, including the neutrophil-to-lymphocyte ratio [34], platelet-to-lymphocyte ratio [35], and delta neutrophil index [36] are widely used in clinical practice. Future studies should aim to integrate existing and new indicators for a comprehensive prediction of endometriosis in women.

The study’s cross-sectional nature limits our ability to establish a causal relationship between SII levels and endometriosis prevalence. Future research with larger samples and prospective designs is warranted. Although adjustments were accounted for several covariates, the potential influence of unknown confounders cannot be discounted. Furthermore, as the study’s participants were exclusively American, further research across various ethnicities is required. Despite these limitations, the study’s strengths lie in a nationally representative sample and an adequate sample size for extensive subgroup analyses.

## Conclusion

The results of this research indicate a significant link between heightened SII levels and an elevated risk of endometriosis, highlighting the potential role of SII as a predictive biomarker for assessing the risk of developing endometriosis. However, to validate these preliminary observations, further research involving extensive, prospective studies is imperative.

## Supporting information

S1 Data (XLS)
